# The Histogenetic Origin of Malignant Cells Predicts Their Susceptibility towards Synthetic Lethality Utilizing the *TK.007* System

**DOI:** 10.3390/cancers16122278

**Published:** 2024-06-19

**Authors:** Fabian Bernhard Pallasch, Vera Freytag, Malte Kriegs, Dennis Gatzemeier, Thomas Mair, Hannah Voss, Kristoffer Riecken, Mona Dawood, Boris Fehse, Thomas Efferth, Hartmut Schlüter, Udo Schumacher

**Affiliations:** 1 Institute of Anatomy and Experimental Morphology, Center for Experimental Medicine, University Cancer Center, University Medical Center Hamburg-Eppendorf, 20246 Hamburg, Germanyu.schumacher@uke.de (U.S.); 2 Department of Diagnostic and Interventional Radiology, Faculty of Medicine, Medical Center—University of Freiburg, 79106 Freiburg Im Breisgau, Germany; 3 Department of Radiotherapy and Radiation Oncology, Hubertus Wald Tumorzentrum–University Cancer Center Hamburg (UCCH), University Medical Center Hamburg-Eppendorf, Martinistrasse 52, 20246 Hamburg, Germany; 4 UCCH Kinomics Core Facility, Hubertus Wald Tumorzentrum–University Cancer Center Hamburg (UCCH), University Medical Center Hamburg-Eppendorf, 20246 Hamburg, Germany; 5 Section Mass Spectrometric and Proteomics, Center of Diagnostics, University Medical Center Hamburg-Eppendorf, 20251 Hamburg, Germany; 6 Research Department Cell and Gene Therapy, Department of Stem Cell Transplantation, University Medical Center Hamburg-Eppendorf, 20246 Hamburg, Germany; 7 Department of Pharmaceutical Biology, Institute of Pharmaceutical and Biomedical Sciences, Johannes Gutenberg University, Staudinger Weg 5, 55128 Mainz, Germany; 8 Department of Medicine, Medical School Berlin, Mecklenburgische Strasse 57, 14197 Berlin, Germany

**Keywords:** chemotherapy, drug resistance, suicide gene transduction, herpes simplex virus thymidine kinase, ganciclovir, functional kinome profiling

## Abstract

**Simple Summary:**

The efficacy of killing human cancer cells with a modified herpes simplex virus thymidine kinase TK.007/ganciclovir (GCV) system was investigated in malignant cells of different histogenetic origin. The aim was to determine whether different histogenetic origins of cancer cells in themselves influence their reaction towards an approach of synthetic lethality, which theoretically should be toxic in a similar range independently of the cell type. Fifteen malignant human cell lines were transduced with a lentiviral vector to stably express the *TK.007* gene and cell proliferation assays under GCV. Among *TK.007*-expressing cell lines, lymphoma and leukemia cells were more susceptible to killing than solid cancer cells, while osteosarcoma and melanoma cells exhibited an intermediate susceptibility. This study highlights that the histogenetic origin of malignant cells strongly influences their susceptibility towards cytotoxic agents, with leukemias and lymphomas being more sensitive than solid cancer cells.

**Abstract:**

Background: Remarkable differences exist in the outcome of systemic cancer therapies. Lymphomas and leukemias generally respond well to systemic chemotherapies, while solid cancers often fail. We engineered different human cancer cells lines to uniformly express a modified herpes simplex virus thymidine kinase TK.007 as a suicide gene when ganciclovir (GCV) is applied, thus in theory achieving a similar response in all cell lines. Methods: Fifteen different cell lines were engineered to express the *TK.007* gene. XTT-cell proliferation assays were performed and the IC_50_-values were calculated. Functional kinome profiling, mRNA sequencing, and bottom-up proteomics analysis with Ingenuity pathway analysis were performed. Results: GCV potency varied among cell lines, with lymphoma and leukemia cells showing higher susceptibility than solid cancer cells. Functional kinome profiling implies a contribution of the SRC family kinases and decreased overall kinase activity. mRNA sequencing highlighted alterations in the MAPK pathways and bottom-up proteomics showed differences in apoptotic and epithelial junction signaling proteins. Conclusions: The histogenetic origin of cells influenced the susceptibility of human malignant cells towards cytotoxic agents with leukemias and lymphomas being more sensitive than solid cancer cells.

## 1. Introduction

The considerable difference in the success rate of systemic therapies such as chemo-, antibody- and/or immunotherapy between hematological neoplasia (leukemias and lymphomas) on the one hand and solid tumors (cancers) on the other is striking. While many entities of the first category can in principle and will in practice be cured by these systemic treatments, widely metastasized cancers will not be cured, and most cancer patients die because of these metastases [1]. It has been hypothesized that this difference is primarily due to the principal architecture of the different tissues. Cancers form tissue aggregates via intercellular structural junctions such as desmosomes, tight junctions, and gap junctions. All these junctions plus the composition of the extracellular matrix and the absence of a functional lymphatic system within cancers leads to an increased intra-tumoral fluid pressure and solid stress [2,3,4]. This pressure limits access of antibodies and small molecule drugs into cancers to around 100 µm around blood vessels [5,6]. It is therefore widely accepted that this increased fluid pressure in cancers limits drug penetration into solid cancers, which contrasts with hematological neoplasia such as lymphomas and leukemias, which are by their very nature individual mesenchymal cells, which do not form tissue aggregates as cancers do and thus do not build up an increased fluid pressure [7].

However, innate resistance of the malignant cells could also be part of the differences observed. As anti-neoplastic drugs differ widely between cancers and lymphomas and leukemias it could be argued that any differences in treatment effects observed are due to the specific drugs used. To overcome the different drug (-combinations) used in lymphoma and leukemia on the one hand and solid cancers on the other, human malignant cells were engineered to express a cancer suicide gene, namely a modified herpes simplex virus (HSV) thymidine kinase. Cell suicide can then be synthetically triggered by the pro-drug ganciclovir, which is metabolized by this enzyme into an active chemotherapeutic drug that kills the cells. Since the lethality induced by an introduced gene is synthetic, all modified cells should, at least in theory, present with similar IC_50_ values for cell killing.

Hence, we transduced several human malignant cell lines with a lentiviral vector expressing a resistance gene for G418, GFP, and the thymidine kinase TK.007. TK.007 is a codon-optimized herpes simplex virus thymidine kinase (HSVtk) with the point mutation A168H [8,9]. The TK.007 thymidine kinase was developed as an inducible suicide gene system for the elimination of alloreactive T cells and graft-versus-host disease [9]. The cytotoxicity measurement in our transduced cell lines revealed remarkable differences in susceptibility according to the histogenetic origin of the malignant cell lines indicating that cell lineage-specific resistance mechanisms exist.

## 2. Materials and Methods

### 2.1. Cell Lines, Cell Culture, and Transfection

Human colon carcinoma cell lines HT-29, CaCo-2, PT-457, and HCT-116, human pancreatic carcinoma cell lines PaCa 5061 and BxPc 3, human leukemia cell lines EOL-1 and K562, lymphoma cell lines Ramos and Raji, human osteosarcoma cell line HOS, small cell lung cancer cell line NCI-H69, and melanoma cell lines LOX, MV3, and MEWO were used. All cell lines except PaCa 5061, which was established in house (https:/h/www.cellosaurus.org/CVCL_C886, last accessed on 1 June 2024), were obtained from the ATTC, and all cell lines were regularly checked for their authenticity as a routine measure in our group [10]. All cell lines were transduced with the lentiviral vector LeGO-TK.007-iG2-Neo carrying the modified herpes simplex virus thymidine kinase gene (*TK.007*), eGFP, and a G418 resistance (NeoR) or a vector control without *TK.007* [9,11,12].

Cells were expanded in T-75 flasks, all cell lines were provided with RPMI-1640 medium (Gibco, Paisley, UK), 10% fetal bovine serum (Gibco), penicillin (100 U/mL, Gibco), and streptomycin (100 µg/mL, Gibco), except the PaCa 5061 cells. PaCa 5061 cells required TUM medium, RPMI Glutamax (Gibco), 10% fetal bovine serum (Gibco), penicillin (100 U/mL, Gibco), streptomycin (100 µg/mL, Gibco), EGF (1 µg/mL, R&D Systems, Minneapolis, MN, USA), FGF (1 µg/mL, R&D Systems), transferrin (0.8 mg/mL, Sigma Aldrich, St. Louis, MO, USA), and insulin (1 µg/mL Sigma Aldrich). All transduced cells were treated with geneticin disulfate (G418; 0.83 mg/mL, Carl Roth GmbH & Co. KG, Karlsruhe, Germany) for several weeks to select the transduced cells. The cells were fed and split as required. Cells were grown in a humidified incubator at 37 °C with 5% CO_2_. For long-term storage, cell lines were kept in liquid nitrogen.

### 2.2. Calibration Curves

A calibration curve was established with every cell line to determine the optimal cell number for the following cell proliferation assay with ganciclovir and to rule out influences of the transduction on cell growth. The growth of cells was quantified using a colorimetric XTT assay (Roche, Mannheim, Germany). The cells were plated in concentrations from 300,000 to 4688 cells/mL in a 96-well plate (Cell+ F 96 well plate, Sarstedt, Germany). The cells were incubated for 72 h, and then 50 µL XTT solution were added. The absorbance was measured at 490 nm and 630 nm in a multi-well spectrophotometer (Dynatech MR 3.13 Micro ELISA reader, Dynex Technologies, Ashford, UK) 6 h after incubation at 37 °C in the incubator.

### 2.3. Cell Proliferation Assay with Ganciclovir

The cell counts for cell proliferation assays were based on calibration curves to achieve a good signal level for the XTT-assays. Cells were seeded in the following entities: 20,000 cells/mL: LOX, MV3, K562; 30,000 cells/mL: HCT-116, HT-29, BxPc 3, MEWO, Caco-2, and PT-457 and HOS; 50,000 cells/mL: PaCa 5061. 100,000 cells/mL: NCI-H69, Ramos, Raji, EOL-1. The above-mentioned XTT-assay was utilized to measure proliferation. The cells were detached using trypsin (5 min trypsin/EDTA; Gibco), resuspended with fresh medium, and counted using a Neubauer chamber. Aliquots of 90 µL cell suspension were plated into each well of a 96-well plate (Cell+ F 96 Well culture plate, Sarstedt, Germany), and cells were allowed to settle. After 24 h incubation, 10 µL ganciclovir (2020 Merck KGaA, Darmstadt, Germany) solution was added (range 10 mmol/mL to 10 nmol/mL), yielding a 1/10 dilution of the final ganciclovir solution. Due to their drug response (see Section 3), we added further GCV dilutions for Ramos, LOX, EOL-1, and K562 cells. As negative control, 10 µL PBS were added. Each ganciclovir concentration was tested in quadruplicate and repeated three times independently, yielding 12 values per concentration. The cells were incubated for 72 h and then measured with the XTT-assay after 6 h.

### 2.4. TK.007 Quantification with PRM

For TK.007 protein quantification, all above mentioned cell lines were used. As biological triplicate cells were grown in T175 flasks till 70% abundance, cell pellets were harvested, counted, and stored at −80 °C.

Samples were lysed in 200 μL of SDC/TEAB buffer (sodium desoxycholat/triethylammoniumbicarbonat) at 98 °C for 5 min and sonicated with 10 pulses at 30% intensity. The protein concentration of the samples was determined using the BCA assay according to the manufacturer’s instructions, with bovine serum albumin used as the standard. An aliquot of 50 μg protein was taken up in 100 μL buffer solution. Disulfide bonds were reduced at 60 °C using 1 μL of dithiothreitol solution (DTT, Sigma-Aldrich, Steinheim, Germany), followed by alkylation with 4 μL in the dark at 37 °C for 30 min. Trypsin protease was added to the samples at a ratio of 1:100 and incubated overnight at 37 °C. Formic acid (FA) was added to lower the solution’s pH to inactivate the protease and precipitate the SDC. The mixture was centrifuged at 16,000× *g* for 5 min, and the supernatant was removed. The solvent was removed with SpeedVac (Uniequip, Martinsried, Germany) until the sample was completely dried. Samples were reconstituted in high-performance liquid chromatography (HPLC)-grade water with 0.1% FA (1 μg/μL) and transferred to sample vials.

The quantification of the TK.007 protein was performed using the Parallel Reaction Monitoring (PRM) method. A Thermo Scientific (Waltham, MA, USA) Dionex Ultimate 3000 UPLC with an autosampler, an online desalting column, and a reversed-phase column were used for this purpose. The separation of peptides was achieved chromatographically using a two-buffer system (buffer A: 0.1% FA in water, buffer B: 0.1% FA in acetonitrile (ACN)). A linear gradient from 2% to 35% B over 65 min was used. The HPLC system was coupled to a Thermo Scientific Q Exactive Orbitrap mass spectrometer. It was analyzed in positive ion mode using the Parallel Reaction Monitoring (PRM) method. Ions with 553.32190 m/z and charge 2+ with an isolation window of 2.0 m/z were selected. Ions were accumulated over a time of 200 ms or until the AGC target of 5 × 10^5^ ions was reached. The resolution was set to 17,500.

For PRM measurement, at least one unique tryptic peptide specific to the protein should be selected, ideally with a complete series of y-ions, to ensure precise quantification. Furthermore, no (post-translational) modifications should be present. The peptide VYGLLANTVR was selected for that purpose. Using a synthetic VYGLLANTVR peptide, a calibration curve was calculated (range 0.001 pg/μL to 100 pg/μL). The fragment ions Y6+ to Y9+ were used for quantification using the Skyline software (version 22.2.0.312).

### 2.5. Bottom-Up Proteomics Analysis

For bottom-up proteomics analysis, all cell lines were tryptically digested as described in Section 2.4. The measurements were also performed on the same LC-MS system as the PRM measurements. The samples were analyzed in the data-dependent acquisition (DDA) mode. For each MS1 scan, ions were accumulated for a maximum of 120 ms or until the AGC-Target (2 × 10^5^ ions) was reached. A mass range of 400 to 1300 m/z and a resolution of 120,000 at a m/z of 200 was applied. Peptides with a charge state of 2+ to 5+ above a minimum signal intensity of 1000 within a 1.6 m/z window were isolated and analyzed in top speed mode with a normalized collision energy of 30% in a higher-energy collisional dissociation (HCD) collision cell. MS2 fragment spectra were obtained by accumulating ions over a time period of 60 ms or until the AGC target (1 × 10^5^ ions) was reached. The applied mass range was 380 to 1500 m/z. Already fragmented peptides were excluded from further fragmentation for 15 s.

Raw data spectra were analyzed in Proteome Discoverer 2.4.1.15 against a human FASTA database (downloaded in April 2020, expanded by the viral Thymidin Kinase ID P06479 human Herpesvirus 1 strain SC16, Swissprot) using the SEQUEST algorithm. Peptides with masses between 250 Da and 5000 Da with lengths between 6 and 144 amino acids were considered. Two missed cleavages were allowed and the mass tolerance of fragmentation at the MS2 level was set to 0.02 m/z. Carbamidomethylations of cyteins were set as fixed modifications; loss of methionine acetylation as well as acetylation after loss of methionine were set as dynamic modifications. A false discovery rate (FDR) of 0.01 was applied.

Further data analysis was conducted in Perseus (version 2.0.10.0, The Perseus computational platform for comprehensive analysis of (prote)omics data [13], where the raw data were log(2)-transformed and column-median normalized. A Student’s *t*-test was performed between good responder and bad responder wild-type cells. Ingenuity Pathway Analysis (IPA, version 94302991, QIGEN Bioinformatics, Venlo, The Netherlands) was used to find significantly regulated pathways between good and bad responder wild-type cell lines. The Ingenuity Knowledge Base was used as a reference dataset. For the proteins, a *p*-value cut-off of 0.05 and a fold change cut-off of 1.5 was used. For pathway enrichment, a z-score algorithm analysis was used with IPA to determine where a pathway was upregulated in the first testing group (z > 0) or the second one (z < 0). *p*-values were calculated with Fischer’s exact test according to IPA software protocols.

Heat maps of proteins annotated to significantly enriched pathways of interest were made in the RStudio environment (Version 4.3.0).

### 2.6. Functional Kinome Profiling

As previously stated, the phosphorylated GCV (ganciclovir triphosphate; GCV-TP) acts as the active drug, which is structurally similar to purine triphosphates such as GTP and ATP, which serve as a phosphate donor for various kinases. We studied this potential influence on molecular communication via functional kinome profiling.

The *TK.007*-expressing pancreatic carcinoma cell line PaCa 5061 and the leukemia cell line K562 were chosen as instructive examples for kinase activity determination. Cells from both cell lines were incubated for 2 h with the specific IC_50_ concentrations of GCV (K562 = 7.1 nmol/mL, Paca 50561 = 31,478 nmol/mL) or were left untreated as a control, respectively. A PamStation 12 (UCCH Kinomics Core Facility, UKE, Hamburg, Germany) was used according to the manufacturer’s instructions (PamGene International, ’s-Hertogenbosch, The Netherlands). In brief, STK-PamChip arrays were used for profiling serine-/threonine kinases and PTK-PamChip arrays for tyrosine kinases [2]. Each array contained 140 (STK) or 196 (PTK) individual phospho-site(s) that are peptide sequences derived from substrates for serine-/threonine kinases or tyrosine kinases, respectively. Whole-cell lysates were made using M-PER Mammalian Extraction Buffer containing Halt Phosphatase Inhibitor and EDTA-free Halt Protease Inhibitor Cocktail (Pierce, Waltham, MA, USA). The protein concentration of the lysate was quantified with a BCA assay (Merck KGaA, Darmstadt, Germany). For STK arrays and for PTK arrays, 1 mg and 5 mg of protein, respectively, and 400 mM ATP were applied. Sequence-specific peptide tyrosine phosphorylation was detected with the fluorescein-labeled antibody PY20 (Exalpha, Maynard, MA, USA) and a CCD camera using the Evolve software (Evolve 3.1.0.5, PamGene International). Serine-threonine phosphorylation was detected in two steps, first with anti-phospho-Ser/Thr antibodies during the reaction followed by detection with a secondary antibody (polyclonal swine anti-rabbit immunoglobulin/FITC, PamGene International). After quality control, the final signal intensities were log2-transformed and were used for further data analysis using the BioNavigator software version 5.1 (PamGene International).

### 2.7. mRNA Sequencing

The Paca5061 TK.007, PaCa5061 WT, K562 WT, and K562 TK.007 cell lines were used. The cells were incubated with IC_50_ concentrations of GCV and harvested after 0 and 4 h. For mRNA extraction, the miRNeasy Mini-Kit (Qiagen, Hilden, Germany) was used according to manufacturer instructions, and mRNA quality was assessed using the Agilent 2100 BioAnalyzer system (Agilent Technologies, Palo Alto, CA, USA).

#### 2.7.1. RNA Sequencing Using Next Generation Sequencing (NGS)

NGS was performed by StarSEQ GmbH, JGU, Mainz, Germany. The quality of the RNA samples was checked by the company using a 2100 Bioanalyzer system (Agilent Technologies, Inc.). After the verification of mRNA samples and library preparation using the NEBNext Ultra II Directional RNA Library Prep kit (New England Biolabs, Ipswich, MA, USA), NGS was carried out using the Illumina NextSeq 500 system using 25 Mio paired-end reads (2 × 150 nt). Bioinformatics was applied via StarSEQ GmbH using the STAR Alignment workflow, followed by pairwise comparison with DESeq2 [14].

#### 2.7.2. Ingenuity Pathway Analysis (IPA)

Data obtained from RNA sequencing using NGS were analyzed with IPA software (version 111725566, Qiagen, Inc.). Core analysis was run for all mapped genes (720–650 deregulated genes) with a *p* value < 0.05. The default parameters for the reference set, relationships to include, node types, mutations, and data sources were set for the analysis except for the species and confidence, the human, and the experimental observations, which were set manually.

### 2.8. Statistics

Statistical analysis of the calibration curves and ganciclovir proliferation assay was performed with GraphPad Prism (Version 5.03 for Windows, GraphPad Software, San Diego, CA, USA). A nonlinear regression was not possible for the negative control and wild-type cells due to the lack of any effect of ganciclovir on these cells, therefore displaying no kinetic relationship.

The datasets were used to obtain IC_50_ values for the TK.007 cells with ganciclovir. The statistical analyses were performed with GraphPad Prism. The data were inserted and first transformed to obtain logarithmic x values, then the y values were normalized with the highest values = 100% and the lowest values = 0%. Then, a nonlinear regression with a normalized response and variable slope with the equation was performed. Graphical presentation of the results was performed with GraphPad Prism.

## 3. Results

### 3.1. Calibration Curves

The calibration curves showed no growth impairment due to transfection. However, there were differences in growth between cell lines. Considering these results, we adapted our cell counts and incubation times for the following ganciclovir-XTT-assay. The spread on cell counts was from 20,000 (LOX, MV3, K562) to 100,000 (NCI-H69, Ramos, Raji, EOL-1). The other cell counts are mentioned above.

### 3.2. Cell Proliferation Assay with Ganciclovir

**Figure 1:**
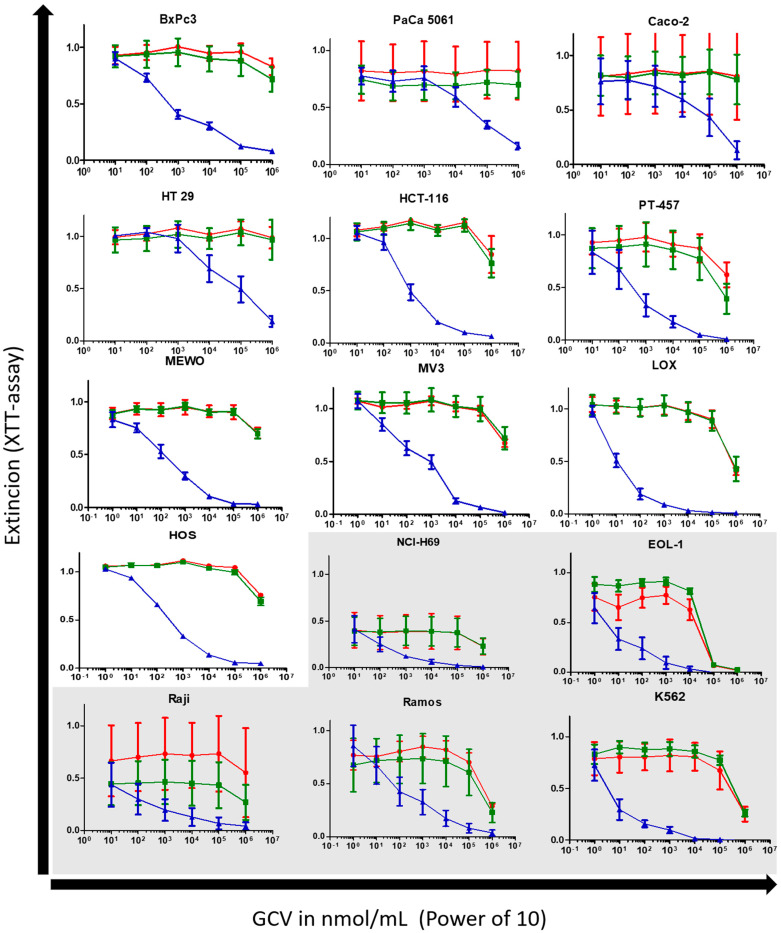
XTT cell proliferation assays are based on 15 different cell lines, wild type (red), negative control (green), and TK.007 (blue) cells. Graphs on the white background are growing adhesive/solid within cell culture, and vice versa the light grey background indicates nonadhesive/suspension growth.

Of all 15 cell lines of different histogenetic origin, leukemia cell lines generally showed the greatest susceptibility towards GCV treatment. Comparing the curve progression in cell proliferation assays, the same reaction pattern to GCV in wild-type and negative-vector-only control cells was seen, indicating no difference in susceptibility of these two cell lines to GCV through the transduction (see Figure 1). The *TK.007* expressing cell lines Caco-2, PaCa 5061, and HT-69 displayed submaximal growth inhibitions to GCV at its highest concentration (Figure 1), with XTT extinction values at around 0.175 (PaCa 5061 at 1 mmol/mL). In the other cell lines, 1 mmol/mL induced an almost complete cessation of cell proliferation with XTT extinction values approaching 0 (see Figure 1).

**Figure 2:**
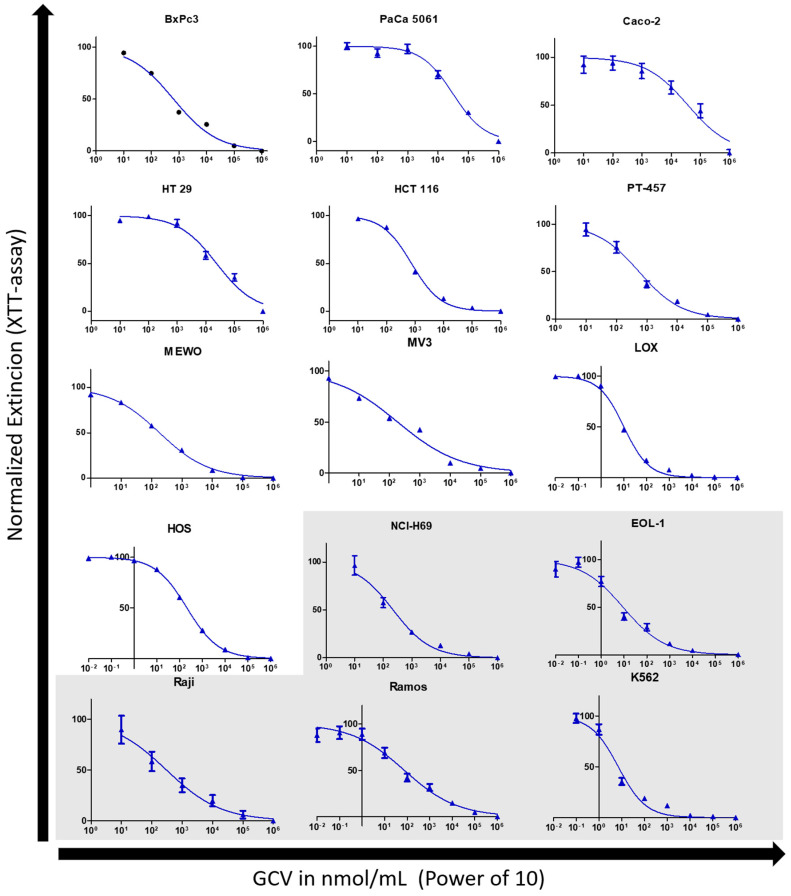
TK.007 response to GCV normalized with a nonlinear curve fit. Graphs on the white background are growing adhesive/solid within cell culture, and vice versa the light grey indicates nonadhesive/suspension growth. Except for LOX, cells with suspension growth display a greater susceptibility to GCV treatment.

The wild type and the negative-vector-only control of the K562 and especially of the EOL-1 cell lines showed a reaction to GCV which is nearly in the range of the TK.007 cells of the other cell lines (i.e., PaCa 5061). The TK.007 K562 and EOL-1 cells yielded IC_50_ values under 10 nmol/mL GCV (Table 1, Figure 2). The lowest value of the leukemia cell lines was yielded with K562 cells with an IC_50_ = 7.2 nmol/mL (95% CI = 5.2–9.7 nmol/mL). The highest IC_50_ value in the leukemia group was observed in EOL-1 cells with an IC_50_ = 9.1 nmol/mL (95% CI = 5.7–14.5 nmol/mL). The highest IC_50_ value from the lymphoma group was noted in Raji cells with an IC_50_ = 305.7 nmol/mL (95% CI = 131.4–711.1 nmol/mL), the other lymphoma cell line RAMOS had an IC_50_ = 63.5 nmol/mL (95% CI = 42.7–131.9 nmol/mL). The transduced melanoma cells MEWO, MV3, and LOX reached IC_50_ values near the lymphoma and leukemia cell lines with LOX at IC_50_ = 10.4 nmol/mL (95% CI = 9.3–11.6 nmol/mL), MV3 with IC_50_ = 189.4 (95% CI = 155.6–230.7 nmol/mL), and MEWO with IC_50_ = 183.990 (95% CI = 152.1–222.6 nmol/mL) (see Table 1).

BxPc3 cells showed the lowest IC_50_ of 634.7 nmol/mL (95% CI = 532.6–756.2 nmol/mL) for the classical cancer cell lines, whereas the highest value was obtained from the PaCa 5061 cells with an IC_50_ = 31,473 nmol/mL (95% CI = 23,345–42,444 nmol/mL) (see Table 1). While these two cell lines grew adherent, the semi-adherent growing colon cancer cell line PT-457 cells reached values below BxPc 3 cells with an IC_50_ = 552.5 nmol/mL (95% CI = 376.2–811.4 nmol/mL).

### 3.3. TK.007 Quantification with PRM

The peptide concentration was calculated from the regression line of the calibration. Two different approaches were used for protein quantification. Initially, a linear regression was employed within the range of 0.250 pg/μL to 100 pg/μL. As a second approach, a forced intercept at 0.050 pg/μL was used. The intercept was very high in the unforced method, causing values that were significantly higher than those of comparable samples in the calibration series. By forcing the intercept, the values in the high concentration range became slightly less accurate, as the difference relative to the calibration points was greater.

The calculated concentrations are presented comparatively for both methods. In Table 2, using the example of the Y8+ ion, which represents the fragment ion with the highest signal intensities. The highest concentrations were found for NCI-H69 TK (Y8+ forced = 107.8 pg/μL and Y8+ linear = 94.7 pg/μL) and HCT-116 TK (Y8 + forced = 107.5 pg/μL and Y8+ linear = 94.5 pg/μL). The lowest concentration was found for Paca 5061 TK (Y8 + forced = 0.11 pg/μL and Y8+ linear = 2.35 pg/μL). The corresponding negative control and the wild type showed higher concentrations (negative control: Y8 + forced = 0.12 pg/μL and Y8+ linear = 2.5 pg/μL and wild type: Y8 + forced = 0.8 pg/μL and Y8+ linear = 2.9 pg/μL).

### 3.4. Bottom-Up Proteomics

**Figure 3:**
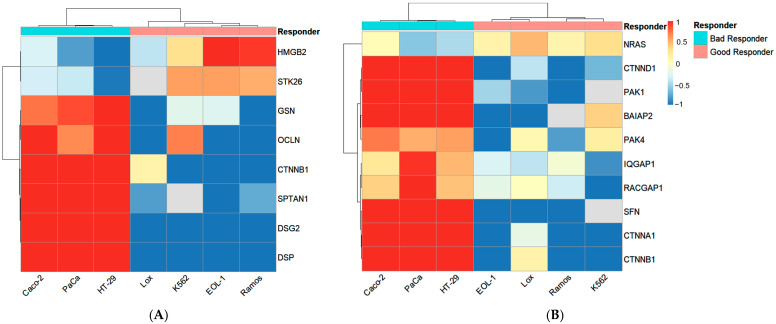
Heat map showing relative abundances of proteins belonging to apoptotic execution phase (**A**) and epithelial adherens junction signaling (**B**). Only significantly regulated proteins (*p*-value < 0.05, fold change > 1.5) between good and bad responder wild-type cell lines are shown. Protein abundances were row-mean normalized over all samples. Red indicates higher protein abundances in a sample compared to the mean, while blue shows lower protein abundances compared to the mean. The samples were annotated according to their respective cell line and response group. Distances on column trees as well as row trees were based on Pearson correlation.

Figure 3 shows two pathways which were found to be significantly enriched between good and bad responder wild-type cell lines via IPA analysis: apoptotic execution phase and epithelial adherens junction signaling. IPA assigned 8 proteins to the apoptotic execution phase and 10 proteins to epithelial adherens junction signaling. Both pathways were upregulated in bad responder cell lines.

Notable proteins are the catenins CTNNB1, which appears in both pathways, and CTNNA1 and CTNND1, which are part of Epithelial Adherens Junction Signaling. All catenins are significantly upregulated in the bad responder wild-type cell lines.

Another interesting protein in this context is PAK1, which interacts with CTNNB1 and has already been described as a potential target for prostate cancer [15].

### 3.5. Functional Kinome Profiling

**Figure 4:**
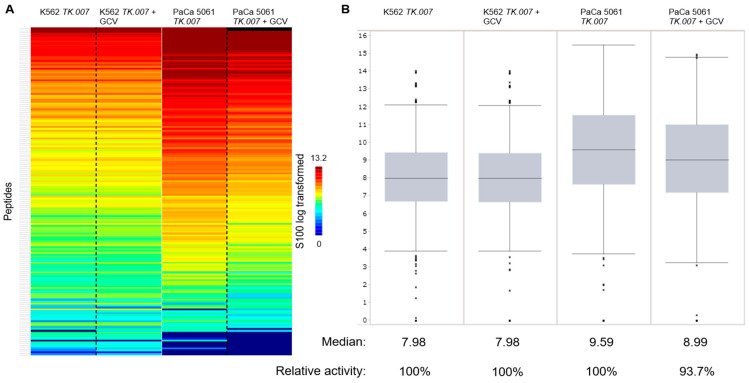
Overall protein tyrosine kinase activity. (**A**) The heat map shows the peptide phosphorylation (the S100_log transformed values are depicted). The two columns on the left compare K562 TK.007 with K562 TK.007 treated with GCV, and the columns on the right compare the PTK activity in PaCa 5061 TK.007 cells with and without GCV. (**B**) Box plot showing differences in overall kinase activity in K562 cells (for technical background [16]).

**Figure 5:**
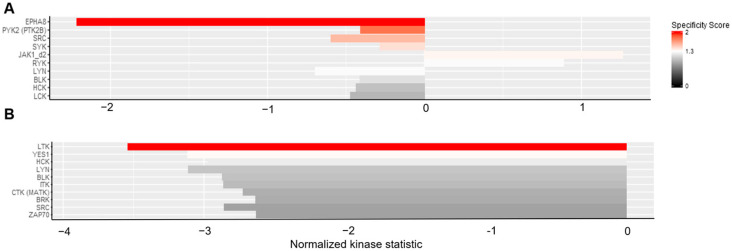
PTK upstream kinase analysis. The upstream analysis of the PTK revealed altered kinase activity after GCV treatment (normalized kinase statistic (log2) < 0: lower kinase activity in inhibitor treated sample; specificity score (log2) > 1.3; white to red bars: statistically significant changes). (**A**) K562 TK.007 vs. K562 TK.007 + GCV. (**B**) PaCa 5061 TK.007 vs. PaCa 5061 TK.007 + GCV. The upstream analysis revealed the altered kinase activity after GCV treatment; the strongest decrease was seen in (**A**) EPHA8 (EPH receptor A8) activity in K562 cells. (**B**) A decrease in LTK (leukocyte receptor tyrosine kinase) activity in PaCa 5061 cells.

**Figure 6:**
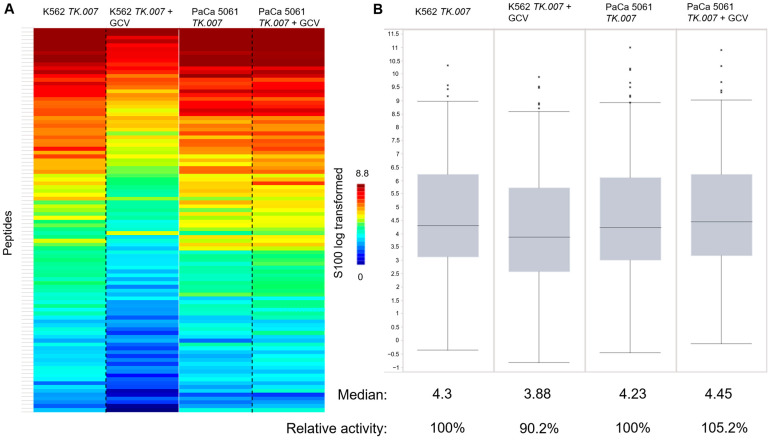
Overall serine-/threonine kinase activity. Overall protein tyrosine kinase activity. (**A**) The heat map shows the peptide phosphorylation (the S100_log transformed values are depicted). The two columns on the left compare K562 TK.007 with K562 TK.007 treated with GCV, and the columns on the right compare the PTK activity in PaCa 5061 TK.007 cells with and without GCV. (**B**) Box plot showing differences in overall kinase activity in K562 cells.

**Figure 7:**
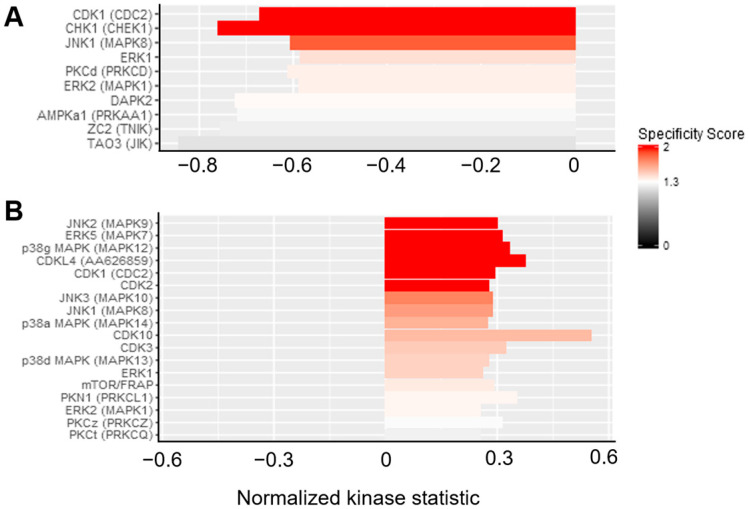
STK upstream kinase analysis from. The upstream analysis of the STK revealed altered kinase activity after GCV treatment (normalized kinase statistic (log2) < 0: lower kinase activity in inhibitor treated sample; specificity score (log2) > 1.3; white to red bars: statistically significant changes). (**A**) K562 TK.007 vs. K562 TK.007 + GCV and (**B**) PaCa 5061 TK.007 vs. PaCa 5061 TK.007 + GCV. The strongest decrease was seen for (**A**) CDK1, CHEK1, and MAPK8 activity in K562 cells, and (**B**) shows an increase in MAPK7, -9, and -12 activity in PaCa 5061 cells.

The PTK results from the PaCa 5061 cells displayed an overall decrease in tyrosine kinase activity due to GCV treatment, see Figure 4. In the upstream kinase analysis, the activity from LTK (leukocyte tyrosine kinase) and YES1 were significantly reduced (specificity score > 1.3), see Figure 5. The STK results showed a small decrease in overall activity for the PaCa 5061 (Figure 6). The upstream analysis revealed significant upregulation in numerous kinases. The top six upregulated serine/threonine kinases were MAPK9 (Mitogen-activated protein kinase), MAPK7, MAPK12, CDKL4 (cyclin-dependent-kinase-like 4), CDK1 (cyclin-dependent-kinase 1), and CDK2 (cyclin-dependent-kinase 2) (Figure 7).

In the PTK of the K562 cells, almost no visible effect in the overall activity of any of the kinases between the transduced and the control group was observed (Figure 4). In detail, the activity of several kinases was significantly altered, the upstream analysis showed a decrease in EPHA8 (ephrin type-A receptor 8), PTK2B (protein tyrosine kinase 2B), SRC, and SYK (spleen tyrosine kinase) activity and JAK1_d2 (Janus kinase d2) displayed increased activity, all with a specificity score > 1.3, see Figure 5. In the STK of the K562 cells, the decrease in overall activity was strong (Figure 6). Upstream kinase analysis showed significant (specificity score > 1.3) downregulation of four kinases, CDK1, CHK1, MAPK8, and ERK1 (Figure 7).

### 3.6. mRNA Sequencing

**Figure 8:**
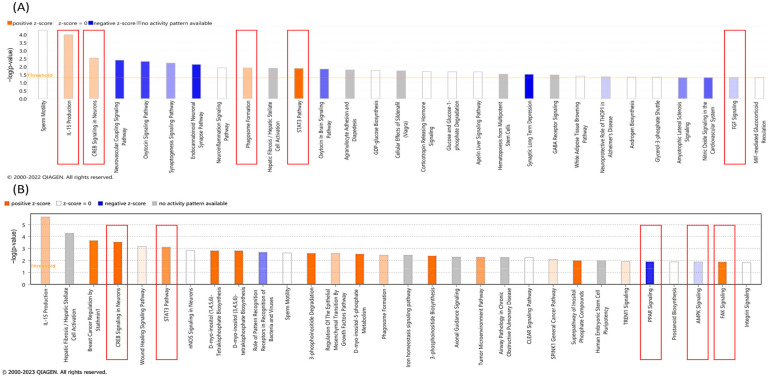
Ingenuity pathway analysis for K562 TK.007 (**A**) and WT (**B**) after treatment with the IC_50_ concentration of GCV. mRNAs responsible for IL production, CREB signaling, STAT3 pathway activation, and phagosome pathway activation were upregulated, while mRNAs for the oxytocin signaling pathway and FGF signaling were inhibited upon treatment [17].

To analyze the molecular effects induced by GCV in *TK.007*-expressing cells, RNA sequencing was performed. Core analysis with IPA identified several canonical pathways in K562 wild-type and *TK.007*-expressing cells upon treatment with GCV. *TK.007*-expressing K562 cells showed an upregulation of mRNA responsible for IL production, CREB signaling, STAT3 pathway activation, and phagosome pathway activation, while mRNAs for the oxytocin signaling pathway and FGF signaling were inhibited upon treatment (Figure 8). On the other hand, K562 wild-type cells showed upregulated mRNA for IL production, STAT3 pathway activation, FAK signaling, and activation of some biosynthesis pathways, while PPAR signaling and AMPK signaling were inhibited upon treatment (Figure 8).

**Figure 9:**
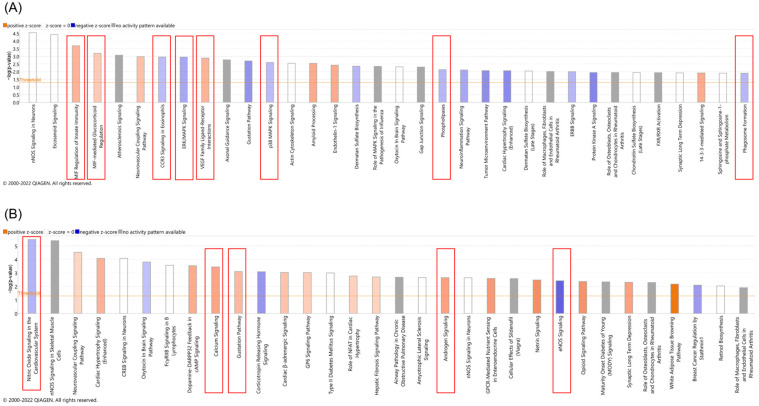
Ingenuity pathway analysis for PaCa 5061 TK.007 (**A**) and WT (**B**) after treatment with GCV (IC_50_ concentration). mRNAs responsible for MIF regulation of innate immunity, MIF-mediated glucocorticoid regulation, and VEGFR family ligands receptor interactions were upregulated, while mRNAs of CCR3 signaling, ERK/MAPK signaling, p38 MAPK signaling, and ERBB signaling, phospholipases, and phagosome formation were downregulated.

Several canonical pathways were activated on the transcriptome of PaCa5061 cells transfected with *TK.007* followed by GCV treatment, such as MIF regulation of innate immunity, MIF-mediated glucocorticoid regulation, and VEGFR family ligands receptor interaction, while others were inhibited, such as CCR3 signaling, ERK/MAPK signaling, p38 MAPK signaling, and ERBB signaling, phospholipases, and phagosome formation. In the PaCa5061 wild-type cells, calcium signaling, the gustation pathway, and androgen signaling were activated upon treatment, but nitric oxide signaling and eNos signaling were predicted to be inhibited (Figure 9).

**Figure 10:**
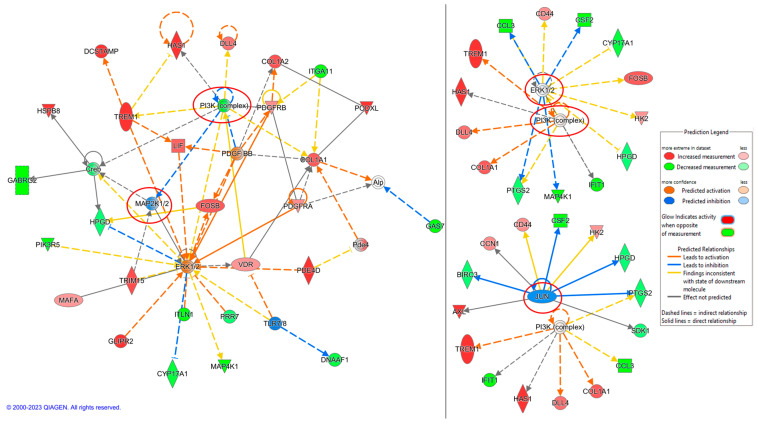
Network analysis of K562 *TK.007*-treated cells. *JUN*, the *PI3K* complex, and *ERK1/2* were affected. The top inhibited network was the MAP2K1/2 and PI3K complex in the core of the network.

**Figure 11:**
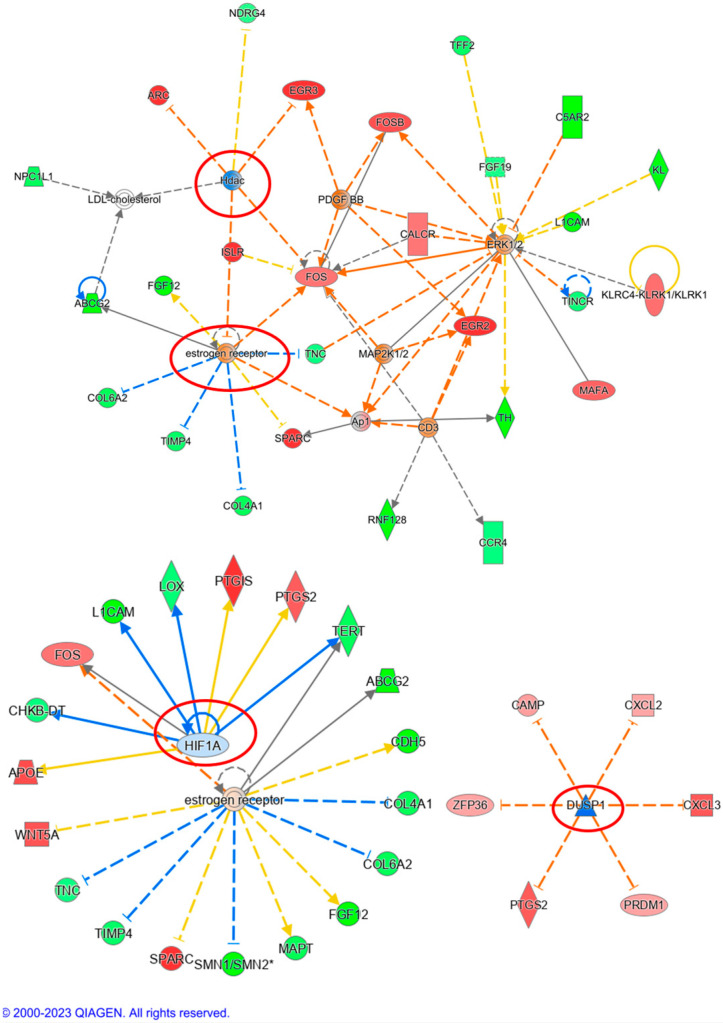
Network analysis of PaCa 5061 *TK.007*-treated cells. The top affected networks were *ESR* and *ERK1/2*, while the top influenced regulators were *HIF1A*, estrogen receptors, and *DUSP4*.

**Figure 12:**
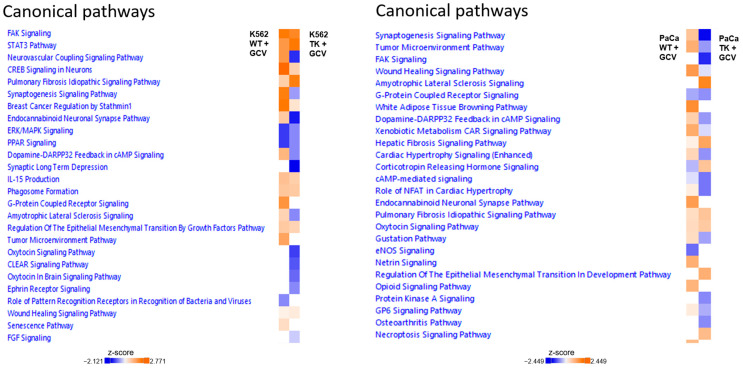
Comparison analysis of the canonical networks showed different activation patterns for *TK.007*-expressing and wild-type cells treated with GCV. Change in status (inhibition to activation or vice versa) of 15 pathways between the K562 wild-type and K562 TK.007 cells (both after treatment), and 21 pathways changed status between PaCa5061 wild-type and PaCa5061 TK.007 cells.

IPA can identify networks and upstream regulators for the uploaded deregulated genes. The most likely upstream regulator of the transcriptome of K562 TK.007-treated cells showed *JUN*, the *PI3K* complex, and *ERK1/2* to be affected. At the same time, network analysis displayed the top network, with the *MAP2K1/2* and *PI3K* complex in the core of the network being inhibited (Figure 10). In PaCa 5061 TK.007 cells treated with GCV, *HIF1A*, estrogen receptors, and *DUSP4* were the top upstream regulators, while the top network displayed *ESR* and *ERK1/2* as affected (Figure 11). The comparative analysis exhibited differences in canonical pathways between the wild-type and transduced cell lines treated with GCV (Figure 12). The status from inhibition to activation or vice versa of 15 pathways changed between the K562 wild-type and K562 TK.007 cells, while 21 pathways changed between PaCa5061 wild-type and PaCa5061 *TK.007*-expressing cells.

## 4. Discussion

Our initial hypothesis was that synthetic lethality induced via transduction of human malignant cells of different histological origins, including lymphomas, leukemias, melanomas, colon, and pancreatic adenocarcinomas, with a suicide gene would result in similar cellular responses. Thus, the malignant cells were transduced via a lentiviral vector to express modified herpes simplex virus thymidine kinase (TK.007) and challenged with ganciclovir, a prodrug that is activated into a chemotherapeutic agent by TK.007. To our surprise, not only the TK.007-expressing cells displayed different kinetics, but so did the wild-type and negative control cells. If the wild-type and negative control cells showed some sensitivity to treatment with GCV, the associated *TK.007* transduced cells displayed a very high susceptibility to GCV.

The etiology of these differences in the cell proliferation assays remains elusive, a plethora of factors likely contributed to the variety of responses to induced cytotoxicity. These effects may evolve around some sort of internal susceptibility to GCV. This finding therefore refutes the original hypothesis that cells of all linages should react uniformly towards the introduction synthetic lethality. For further experimental use of this system in mice, it is therefore necessary to adapt the ganciclovir concentration to the cell line-specific IC_50_ value.

We successfully quantified TK.007 and found no correlation between the amount of TK.007 within the cells and the response to treatment, indicating that the amount of the enzyme is not the rate limiting step for the cytotoxicity. During the quantification of the tryptic peptide, it was identified in all samples, including both wild types and the negative control. Given that this involves a viral thymidine kinase, it should not be present in human tumor cell lines. It can be ruled out as a similar peptide, since a variety of fragment ions (Y1+ to Y9+ and B2+ to B4+) were detected. Contamination during sample preparation was unlikely, as it was present in all samples, and the peptide’s concentration was relatively consistent across all wild-type and negative control samples (0.05–0.45 pg/μL). Theoretically, these signals could also be explained by carry-over during liquid chromatography. However, randomization of the samples during analysis argues against this hypothesis. The peptide was only found in viral thymidine kinases. Another explanation is the integration of viral DNA into the human genome since approximately 45% of human DNA consists of foreign DNA [18]. Regarding viral thymidine kinase, there are no references confirming this assumption. However, the modified TK.007 protein and several known thymidine kinases of human herpesvirus 1 do not differ in this peptide, suggesting that it was likely the unmodified DNA of a herpesvirus.

The transcriptome of the transfected cells differed after treatment with GCV. There were also significant alterations in tyrosine kinases such as FAK (focal adhesion kinase), which is a cytoplasmic tyrosine kinase that plays a role in integrin-mediated signal transductions. FGF signaling was one of three transduction pathways: RAS/MAP kinase, which is a tyrosine kinase, PI3K/AKT (serine/threonine kinase), or PLCγ (phospholipases) [19,20]. This finding matches our functional kinome profiling. From the canonical pathways, there was always a difference in the pathways between the transduced cell and the non-transduced one after GCV treatment. However, the differences were remarkable in the human pancreatic carcinoma PaCa 5061 cell line in comparison to leukemia K562 cells. Other important cancer-related molecules and pathways were inhibited upon GCV treatment, for example, HIF1A, MAP2K1/2, PI3K, JUN, and HDAC.

Considering that small molecules such as imatinib act via molecular mimicry of ATP by blocking the kinase domain of BCR-ABL, we further hypothesized that, due to its molecular structure, ganciclovir might interact with human kinases, transcriptome, and proteome, especially with GTP-dependent pathways [21]. Pamstation 12 analyses the phosphorylation of different peptide sequences. From the alterations in the phosphorylation patterns, it was possible to calculate quantitative and qualitative alterations of kinase activities, thus being a tool to search for unknown actions of GCV on human kinases. Hence, the PTK assay revealed numerous alterations in the phosphorylation pattern of specific peptides, enhancing the quality of the upstream analyses, which revealed the above-mentioned significant alterations in tyrosine kinases (Figure 3 and Figure 4). The few significant altered peptides in both STKs weakened their experimental value. Nevertheless, a few significant alterations were found (Figure 5 and Figure 6). The downregulations of LTK, EPHA8, and PYK2 tyrosine kinases were likely to influence survival and proliferation. Especially interesting was the common suppression in the activity in SRC family kinases (SFKs), which are also targets of other tyrosine kinases such as EPHA8 [22]. The significantly altered SFKs are YES1 in PaCa 5061 and LYN and SRC in K562 cells. In combination with further nonsignificant suppressions of SFKs (i.e., HCK and LCK) the kinome data indicate a suppression of this protein family through ganciclovir. The SFKs contain eight members (YES, SRC, BLK, FGR, FYN, LYN, HCK, and LCK), they are further separated into two groups, SRC-A (SRC, YES, FGR, and FYN) and SRC-B (LYN, LCK, HCK, and BLK). In mammals, the SRC-A group, except FGR, is ubiquitously expressed; the SRC-B group expression varies within different tissues. Due to their family resemblance, they can be looked at as a collective and not as individual kinases [23,24]. SFKs are implicated in numerous cellular pathways for cell growth, adhesion, division, survival pathways, and migration. The SRC family members display considerable crosstalk, a complex expression pattern, a promiscuous substrate pattern, and have redundant functions; therefore, the integration of SFKs in normal cell biology and cancer signaling remains elusive and is not within the scope of this paper [25]. Their reciprocal influence with cellular junctions, such as integrins and E-Cadherin, makes them an interesting factor in the observed differences within the cell proliferation assays, as SRC inhibition was shown to modulate adhesion strength via E-Cadherin in A431 (derived from squamous cell carcinoma) cells and to reduce motility and invasion [26]. SFKs re-induce E-Cadherin and the above-mentioned pro-apoptotic properties of E-Cadherin re-expression at first glance seem contrary to our results, but this effect is only relevant in cell lines descendent from E-Cadherin-expressing cell lines. The cell lines that are still adhesive are not likely to suffer as much from E-Cadherin re-expression, because E-Cadherin was there all along, and apoptosis evasion might not ground on the E-Cadherin/Bcl-2 link in these cell lines resulting in not as much of a protective phenotype of E-Cadherin but instead as a vulnerability through E-Cadherin suppression and re-expression. This is especially relevant for the differences seen within the solid cancer types such as with HT-29 (IC_50_ = 23,773 nmol/mL) and HCT-116 (IC_50_ = 755.1 nmol/mL), as HT-29 is known for a higher E-Cadherin expression compared to HCT-116 cells as seen in Western blots with an E-Cadherin antibody [27]. In another study with BxPc3 cells, SRC inhibition with dasatinib was found to restore E-Cadherin levels [28].

The relatively higher presence of apoptotic execution phase proteins and epithelial adherens junction signaling proteins in wild-type cells of the bad responder group supports the hypothesis that the epithelial phenotype possesses protective properties. From a logical standpoint, an increase in apoptotic execution phase proteins would typically be expected to result in more apoptosis. However, cells exhibiting an epithelial phenotype appear to have acquired a resistance to cell death. Of special interest is the dysregulation of catenins (CTNNB1, CTNNA1, and CTNND1). A study using a tumor metastasis PCR array found CTNNA1 to be upregulated in PDAC cancer metastasis [29], while another study on colorectal cancer found the downregulation to be associated with a more aggressive phenotype [30]. The role of CTNNB1 (a.k.a, beta-catenin) is known to be complex, on the one hand mediating the gene expression of the WNT-signaling pathway and on the other hand playing a crucial role in stabilizing cell–cell adhesion [31,32].

As another observation, the cytotoxic effect of ganciclovir on the *TK.007*-transfected cells seem, to a certain extent, proportional to the growth kinetics evaluated in calibration curves prior to the cytotoxicity experiments. Cells with shorter cell cycles had to be diluted higher to accommodate the faster cell number increase in the following experiments. Ganciclovir is a prodrug guanine analog and only if it has a triphosphate residue is it incorporated into the DNA, resulting in DNA instability. With this mode of action, the integration of ganciclovir into the DNA is dependent on the speed of cell cycles. The quicker the S-phase is reached, the more ganciclovir is integrated into the DNA, which is a well-known observation in chemotherapy [33].

Comparing the slow-growing EOL-1 cells with the fast-growing K562 cells, it is obvious that factors other than cell cycle progression must contribute to the cytotoxic effect of ganciclovir. Both fast and slow cycling cell lines yielded IC_50_ values in the same concentration range with no significant differences, implicating that other diverse influence factors, such as molecular background (susceptibility and protecting factors, e.g., intercellular junctions, expression of kinases, control of dNTP equilibrium, etc.) and/or metabolism, may take part in the effectivity of ganciclovir application.

In a previous preclinical toxicity study, the effect of TK.007 in brain tumors was investigated. *TK.007*-expressing normal brain cells survived the GCV treatment, and the treated rats displayed no differences in behavior experiments compared with the control group [11]. This observation supports the theory that normal cells are not as affected by ganciclovir as malignant cells are. However, one must consider that neurons are classified as permanent cells without participation in the cell cycle; hence, the effect of GCV is minimalized by the very nature of these cells. At the other side of the cell cycle spectrum are labile cells, such as hematopoietic stem cells, which continuously proliferate. As myelosuppression is a known side effect of GCV treatment [34,35,36], this indicates that normal cells may be affected by ganciclovir treatment. This known general effect on hematologic cells lines up with our results, as the strongest effect was seen in cells of hematopoietic origin.

To address possible susceptibility factors towards chemotherapy-induced apoptosis, we looked at the cells of origin of the malignancies investigated. Looking at the origin of the malignant cell background, the spectrum ranges from liquid cancers with single isolated or small oligo cell aggregates, forming no solid cancer strands surrounded by a tumor stroma on the one hand, and solid cancers forming large cellular aggregates surrounded by connective tissue stroma on the other hand [37]. The liquid spectrum is represented by leukemias and lymphomas, and the solid ones by colorectal and pancreatic carcinomas. In between this spectrum are sarcoma and melanoma cell lines, with features of liquid cancers and solid cancers; in particular, the latter cell line forms no structural cell–cell junctions typical for cancers [38]. Comparing this phenotypic characterization, with their ganciclovir response, it becomes apparent that the response at least correlates with their adhesive spectrum. The spread of response within the cell lines with a similar phenotype (e.g., HT-29 IC_50_ = 23,775 nmol/mL vs. HCT-116 IC_50_ = 755.1 nmol/mL) could be mainly explained by differences in growth speed and internal degree of epithelial differentiation/degree of EMT. This hypothesis is underlined with the previously described higher expression of the mesenchymal marker vimentin in HT-29 cells compared to HCT-116 where vimentin expression is absent [39]. The key epithelial marker E-Cadherin can be directly linked to apoptosis. It was shown that re-expression of E-Cadherin promotes apoptosis, and loss of E-Cadherin increases the anti-apoptotic Bcl-2 [40,41].

In comparison to the other basic suicide gene system, such as the Cytosine deaminase/5-fluorocytosine (CD/5-FU) system, the TK.007/GCV system displays less direct bystander effects [42,43], making our system favorable for the examination of tumor-associated cells and the tumor-associated matrix. Further in vivo studies should consider this multifactorial susceptibility of different entities to ensure that the dosage of GCV is sufficient and optimized for optimal experimental success and to minimize adverse reactions to the GCV treatment, such as the above-mentioned myelosuppression. Further in vivo studies in mice could investigate IC_50_-adapted GCV dosage regarding treatment efficacy, effect on the tumor microenvironment, and occurrence of adverse events.

Melanocytes develop from melanoblasts within the neural crest. During embryogenesis the melanoblasts migrate mainly through the dorsolateral route all over the skin, showing that proliferation, detachment, and motility are in the very nature of these cells [44]. Noticeably, the fast-growing melanoma cell line Lox showed a cytotoxic ganciclovir response in the same order of magnitude as the leukemia cell lines leading to the hypothesis that the epithelial character as such has an important protective effect against ganciclovir-induced apoptosis. The MEWO and MV3 melanoma cells had IC_50_ values around 20–35-times higher (184 nmol/mL and 189.4 nmol/mL) than leukemia cells and the LOX melanoma cell (10.4 nmol/mL), but the IC_50_ values were considerably lower compared to the values of the colon and pancreatic cancer cells (Table 1).

According to the IC_50_ values, three major clusters could be identified which fit surprisingly well with the embryological origin of the malignant cells. An endodermal-, ectodermal-, and mesodermal-derived cluster appeared. Endodermal-derived cells have their background directly in the endodermal group and form “real epithelial phenotype cells” represented by pancreatic and colon cancer cell lines. They represent a classical epithelial phenotype. The (neuro-) ectodermal-derived cells are from melanoma, while the mesodermal-derived cells are leukemia, lymphoma, and osteosarcoma cells.

## 5. Conclusions

The TK.007 suicide kinase system provides an effective system for malignant cell killing with surprising variability in cellular response to this artificially introduced suicide system. Using the PRM method, we quantified the introduced TK.007 viral thymidine kinase and proved the success of our transduction. In line with its synthetic nature, the quantity of TK.007 did not appear to be critical for therapy response and was therefore not the rate limiting step in killing the malignant cells. This system has the advantage that it allows for the examination and comparison of cellular responses and resistances between entities that are typically not practically comparable due to their differences in clinical therapy regimens. Thus, our comparative approach opens new gates for comparing and finding so-far unknown resistance mechanisms. Despite the synthetic character of the lethality, the reaction of different cancer entities in cell culture displays huge differences, which can be partially explained by influencing kinomics, proteomics, and transcriptomics, but the exact genesis of these differences is most likely extremely complex and multifactorial and needs to be further elucidated.

## Figures and Tables

**Table 1:** IC_50_ values for the *TK.007*-expressing cells are shown, as expected the negative control and the wild-type cells did not yield presentable data, due to lack of response to GCV.

Cell Line	IC_50_ (nmol/mL)	Log IC_50_	R^2^	95% Confidence Interval (nmol/mL)
HCT-116 TK.007	755.1	2.9	0.981	671–849.9
HT-29 TK.007	23,773	4.4	0.900	17,672–31,979
Caco-2 TK.007	37,752	4.6	0.634	18,760–75,971
PT-457 TK.007	552.5	2.7	0.866	376.2–811.4
BxPc3 TK.007	634.7	2.8	0.971	532.6–756.2
PaCa 5061 TK.007	31,478	4.5	0.900	23,345–42,444
NCI-H69 TK.007	222	2.4	0.811	142.9–344.7
LOX TK.007	10.4	1.0	0.989	9.3–11.6
MV3 TK.007	189.4	2.3	0.976	155.6–230.7
MEWO TK.007	184	2.3	0.975	152.1–222.6
RAMOS TK.007	75.1	1.9	0.836	42.7–131.9
Raji TK.007	305.7	2.5	0.575	131.4–711.1
K562 TK.007	7.1	0.9	0.921	5.2–9.7
EOL-1 TK.007	9.1	1.0	0.869	5.7–14.5
HOS TK.007	209.6	2.2	0.996	194.9–225.4

**Table 2:** Calculated TK.007 concentrations; notably, even non-transduced cell lines show some amount of protein, most likely due to background from TK.007 being indistinguishable from other virus thymidine kinases, which are most likely integrated into human DNA since 45% of human DNA is considered foreign (further discussed below within Section 4).

Cell Line	Y8+ Forced (pg/µL)	Y8+ Linear (pg/µL)
Paca WT	0.8	2.94
PaCa NGK	0.12	2.35
Paca TK.007	0.11	2.34
K562 WT	0.08	2.32
K562 NGK	0.18	2.40
K562 TK.007	20.3	19.66
MV3 WT	0.06	2.30
MV3 NGK	0.01	2.26
MV3 TK.007	31.27	29.08
Lox WT	0.16	2.39
Lox NGK	0.12	2.35
Lox TK.007	23.85	22.71
BxPc 3 WT	0.07	2.31
BxPc 3 NGK	0.23	2.45
BxPc 3 TK.007	5.36	6.85
HT 29 WT	0.08	2.32
HT 29 NGK	0.16	2.38
HT 29 TK.007	4.15	5.81
HCT 116 WT	0.08	2.32
HCT 116 NGK	0.31	2.52
HCT 116 TK.007	107.53	94.50
MEWO WT	0.08	2.32
MEWO NGK	0.18	2.4
MEWO TK.007	15.83	15.83
EOL 1 WT	0.14	2.37
EOL 1 NGK	0.15	2.38
EOL1 TK.007	17.45	17.22
PT 457 WT	0.24	2.45
PT 457 NGK	0.32	2.52
PT 457 TK.007	66.06	58.93
HOS WT	0.13	2.36
Hos NGK	0.20	2.42
HOS TK.007	5.69	7.13
Caco WT	0.16	2.39
Caco NGK	0.44	2.62
Caco TK.007	0.84	2.97
Ramos WT	0.18	2.40
Ramos NGK	0.19	2.41
Ramos TK.007	64.26	57.38
Raji WT	0.13	2.36
Raji NGK	0.13	2.36
Raji TK.007	5.34	6.83
NCI H69 WT	0.40	2.59
NCI H69 NGK	0.35	2.54
NCI H69 TK	107.76	94.7

## Data Availability

Upon request, the data can be obtained through correspondence.
